# Health leaders’ perspectives and attitudes on medical assistance in dying and its legalization: a qualitative study

**DOI:** 10.1186/s12910-025-01208-2

**Published:** 2025-05-06

**Authors:** Amanda Yee, Eryn Tong, Rinat Nissim, Camilla Zimmermann, Sara Allin, Jennifer L. Gibson, Madeline Li, Gary Rodin, Gilla K. Shapiro

**Affiliations:** 1 Leslie Dan Faculty of Pharmacy, University of Toronto, Toronto, ON Canada; 2 Department of Supportive Care, Princess Margaret Cancer Centre, Toronto, ON Canada; 3 Department of Applied Psychology and Human Development, Ontario Institute for Studies in Education, University of Toronto, Toronto, ON Canada; 4 Department of Psychiatry, Faculty of Medicine, University of Toronto, Toronto, ON Canada; 5 Global Institute of Psychosocial, Palliative and End-of-Life Care (GIPPEC), University of Toronto and Princess Margaret Cancer Centre, Toronto, ON Canada; 6 Department of Medicine, Faculty of Medicine, University of Toronto, Toronto, ON Canada; 7 Institute of Health Policy, Management and Evaluation, Dalla Lana School of Public Health, University of Toronto, Toronto, ON Canada; 8 Joint Centre for Bioethics, Dalla Lana School of Public Health, University of Toronto, Toronto, ON Canada; 9 Clinical Public Health Division, Dalla Lana School of Public Health, University of Toronto, Toronto, ON Canada; 10 Epidemiology Division, Dalla Lana School of Public Health, University of Toronto, Toronto, ON Canada; 11 Social & Behavioural Health Sciences Division, Dalla Lana School of Public Health, University of Toronto, Toronto, ON Canada

**Keywords:** Medical assistance in dying, End-of-Life care, Health policy, Attitudes, Perspectives, Canada, Qualitative research

## Abstract

**Background:**

Medical Assistance in Dying (MAiD) has transformed health policy and practice on death and dying. However, there has been limited research on what shaped its emergence in Canada and the beliefs and views of health leaders who hold positions of influence in the healthcare system and can guide policy and practice. The objective of this study was to examine health leaders’ perspectives on the factors that led to the emergence of MAiD and explore their attitudes about the legalization of MAiD.

**Methods:**

In this qualitative study, we conducted online semi-structured interviews with health leaders from April 2021 to January 2022. Purposive and snowball sampling techniques were used to recruit health leaders who have expertise and engagement with the delivery of MAiD or palliative and end-of-life care, and who hold positions of leadership relevant to MAiD in their respective organisations. Inductive thematic analysis was used to analyze the transcribed interviews.

**Results:**

Thirty-six health leaders were interviewed. Participants identified six factors that they believed to have led to the introduction of MAiD in Canada: public advocacy and influence; judicial system and notable MAiD legal cases; political ideology and landscape; policy diffusion; healthcare system emphasis on a patient-centred care approach; and changes in societal and cultural values. Participants expressed wide-ranging attitudes on the legalization of MAiD. Some described overall agreement with the introduction of MAiD, while still raising concerns regarding vulnerability. Others held neutral attitudes and indicated that their attitudes changed on a case-by-case basis. Participants described four factors that they considered to have had influence on their attitudes: personal illness experiences; professional experiences and identity; moral and religious beliefs; and, the valence of patient autonomy and quality of life.

**Conclusions:**

This study highlights the wide-ranging and complex attitudes health leaders may hold towards MAiD and identifies the convergence of multiple factors that may have contributed to the legalization of MAiD in Canada. Understanding health leaders’ attitudes and perspectives on the legalization of MAiD may inform stakeholders in other countries who are considering the legalization of assisted dying.

**Supplementary Information:**

The online version contains supplementary material available at 10.1186/s12910-025-01208-2.

## Background

Assisted dying is legal in a growing number of jurisdictions worldwide but remains illegal in most countries in the world [1]. In Canada, assisted dying is referred to as Medical Assistance in Dying (MAiD) and was first legalized in 2016 [2]. The original legislation permitted adults to request MAiD if they met specific criteria including suffering from a “grievous and irremediable medical condition” and a “reasonable foreseeability of natural death” [3]. In 2021, the criterion of having a “reasonable foreseeability of natural death” was removed as a requirement for Canadians seeking MAiD [3], which created a pathway to receive MAiD for individuals suffering solely from mental illness. The legalization of MAiD for mental illness has twice been deferred in this country due to concerns about readiness and is anticipated to become available to patients in March 2027 [3].

Past research has explored the perspectives and attitudes of the public and healthcare providers toward the introduction of MAiD. Most Canadians have expressed support for legalizing MAiD [4–6], while healthcare providers have reported varied levels of support [5, 6]. The growing literature on this subject has examined the knowledge, perspectives, and attitudes of healthcare providers toward MAiD including that of physicians, psychiatrists, nurses, pharmacists, and others [[7–10]. In some studies, healthcare providers have voiced dissatisfaction with, and concerns about, MAiD [11]. Other research has indicated support among other healthcare providers, who viewed MAiD as a form of care that allows patients to exercise their autonomy and reduces the physical and psychosocial suffering of the patient and family [12]. Several reviews have synthesized this research [13–15], often highlighting the complexity of emotions and experiences of those providing MAiD, as well as the challenges for clinical practice. Attitudes towards MAiD may be influenced by multiple factors [5] such as individual religious beliefs [8, 16] or healthcare providers’ clinical experiences [16, 17]. One study that examined the experience of MAiD assessors and providers (i.e., physicians and nurses) regarding legal changes to MAiD highlighted the importance of involving MAiD assessor and providers in policy discussions in advance of legislative changes [18].

To date, there has been no national study on the perspectives and attitudes of Canadian health leaders towards the introduction of MAiD, and there has been limited research on the beliefs of health leaders in relation to the emergence of MAiD in Canada. Health leaders are, by definition, individuals who hold positions of influence in the healthcare system and who can guide policy and practice. The present study included healthcare providers (e.g., MAiD assessors and providers) and leaders who held positions in government, non-profit organizations, or academia. Understanding attitudes among multiple health leader groups (i.e., not exclusively healthcare providers) can elucidate the process that led to assisted dying policies, can inform ongoing discussions about evolving MAiD policy in Canada, and can provide key lessons that other countries can consider in the introduction of assisted dying. In this study, we examined health leaders’ perspectives about what factors shaped the legalization of MAiD, their attitudes towards MAiD, and their views about what shaped their attitudes.

## Methods

### Study design

This qualitative research study was developed as part of a national project that explored health leaders’ perspectives regarding the delivery of MAiD in Canada. The current study focused specifically on health leaders’ perspectives on the factors that led to the emergence of MAiD, and their attitudes about the legalization of MAiD. The study protocol has been published previously [19], and this study followed the Consolidated Criteria for Reporting Qualitative Research (COREQ) checklist (Supplementary Material) [20].

The research team encompassed a range of academic disciplines, occupations, career stages, and personal backgrounds. The team was multidisciplinary with expertise in health policy, health services, bioethics, psychiatry, palliative care, and psychology. They were researchers, clinicians, and health leaders in different stages of their career (i.e., trainees, andearly-, mid-, and senior career researchers). There were male and female members of the research team. The team adopted a neutral stance regarding MAiD [19].

### Study participants

Health leaders included in this study are individuals who have expertise and engagement with the delivery of MAiD or palliative and end-of-life care, and who hold positions of leadership relevant to MAiD in their respective organisations [13]. This encompasses leaders with roles in developing or modifying policies about MAiD including, but not limited to, medical and nursing division heads, program directors and managers, presidents or CEOs of nongovernmental societies or associations, lead government policy analysts, scientists with expert specialization, and chairs of advisory committees on MAiD.

We recruited participants using purposive and snowball sampling. We sampled MAiD health leaders with appropriate expertise who had been involved in developing or implementing MAiD policy, and those who observed this process while holding leadership positions during this period (e.g., individuals who contributed expert advice to inform MAiD legislation or provided guidance for the expert panels on MAiD).

### Data collection

Data collection took place from April 2021 to January 2022. All data was collected online. The interview guide has been previously published as supplementary material in the study’s protocol paper [19]. One research team member (GKS) conducted the interviews. The interviewer did not have an established relationships with participants beyond the initial contact. During the initial contact, the participants were provided a brief explanation of the study’s purpose and invited to participate.

The interview began with collecting sociodemographic information followed by in-depth, semi-structured questions. For example, sample questions included: “what is your attitude towards MAiD”, “can you explain what has shaped your attitude toward MAiD”, and “what do you believe were the factors that allowed for the legalization of MAiD in Canada (why here why now)”. All participants were invited to provide any further comments and ask questions. We conducted individual interviews with Canadian health leaders until data saturation was reached (i.e., no new themes were identified). Interviews were audio-recorded and lasted on average 56 minutes. During the interviews we used respondent validation and checked emerging themes to ensure findings were accurate, valid, and generalizable.

### Data analysis

All interviews were transcribed using a secure transcription service (Temi.com) [21]. Transcripts were verified and de-identified. Inductive thematic analysis was used to analyze the transcribed interviews using NVivo12 software [22]. During analysis, weekly team meetings (AY, ET, GKS) were held to discuss emerging code development by the main reviewer (AY), consolidate key themes, select representative quotations, increase reflexivity, and allow the team to reflect on their positionality. We discussed codes until we reached consensus. To minimize bias, two additional team members (RN and GR) reviewed codes and themes. The diverse composition of our larger research team facilitated collaborative discussions and ongoing reflexivity when developing and interpreting the themes.

### Ethics statement and consent

This study received ethical approval from the University Health Network Research Ethics Board (#19-5518) [19, 23]. Before the interviews commenced, the aims of the interviews were explained and informed consent was obtained from each participant.

## Results

### Participant characteristics

Table 1 outlines the characteristics of study participants. Overall, most participants were between 40 and 69 years old (77%), female (72%), White (83%), and either reported not having any religious beliefs or being agnostic or atheist (53%). Most participants reported holding a health professional role (64%) in addition to administrative (55%), academia (50%), not-for-profit (47%), and government roles (22%).

**Table 1:** Sociodemographic characteristics of participants

Characteristics	No. (%) *n* = 36
**Age (years)**	
20–39	4 (11)
40–69	28 (78)
70–79	1 (3)
Prefer not to answer	3 (8)
**Gender**	
Female	26 (72)
Male	9 (25)
Gender fluid	1 (3)
**Ethnicity**	
White	30 (83)
Asian (i.e., East Asian, South Asian, Indian)	3 (8.5)
First Nations	2 (5.5)
Prefer not to answer	1 (3)
**Religion**	
None, agnostic, or atheist	19 (53)
Christian	10 (28)
Islamic	1 (3)
Jewish	3 (8)
Spiritual	1 (3)
Prefer not to answer	2 (5)
**Region**	
Central Canada	11 (31)
National	10 (28)
Western Canada	6 (17)
Atlantic Canada	5 (14)
Territories	2 (5)
Prairies	2 (5)
**Years of professional experience**	
2–15	8 (22)
16–30	21 (58)
*≥*31	6 (17)
No answer	1 (3)
**Leadership role**	
Health professional	23 (64)
Administrative	20 (55)
Academia	18 (50)
Not-for-profit	17 (47)
Government	8 (22)

^a^ Participants could select multiple options

### Perceived factors leading to the introduction of MAiD

Health leaders described six factors they believed had contributed to the introduction of MAiD in Canada (Table 2).

*Public advocacy and influence.* Many participants believed that public advocacy and organizations played a central role in drawing attention toward the legalization of MAiD and shaping public discourse. They noted that the narratives of those with physical or psychological disabilities humanized the issues and served as a catalyst for social mobilization: “We’re listening to people [and] maybe hearing voices that maybe weren’t heard before, [amongst the] marginalized communities, that includes people with disabilities and chronic illnesses (P23).”

*Judicial system and notable MAiD legal cases*. Many participants highlighted how the legalization of MAiD in Canada stemmed from a series of legal challenges to the criminal code, a process that they contrasted with the introduction of assisted dying in other countries. For example, they pointed to two pivotal legal cases. *Rodriguez v. British Columbia*, which stimulated reflection about the Canadian Charter, and *Carter v. Canada*, which challenged the Criminal Code. These court rulings were perceived to be a key driver of the advancement of MAiD: “I don’t think there was [a] strong [MAiD] movement here in Canada, but because there were individuals like Carter who took this case all the way to the Supreme Court […] We’ve done it backwards compared to other countries (P3).”

*Political ideology and landscape*. Some participants explained that the political beliefs held by the public and the election of a centre-left government in 2015 reflected a common zeitgeist that led to the introduction of MAiD. They described the alignment of widely held liberal values of individual autonomy and personal choice that aligned with the ideologies held by the centre-left federal political party (i.e., the Liberal Party of Canada) and with the arguments made in favour of the introduction to MAiD. Health leaders explained that the introduction of MAiD in Canada occurred around the time that Canada elected the centre-left Liberal Party of Canada: “…I think we only got [MAiD] because the Liberals got in, we wouldn’t have had it if the [centre-right] Conservatives stayed in (P36).” Even though MAiD was legalized through the decision of the Supreme Court of Canada, some participants believed the political ideologies and landscape at the time were related to the introduction of MAiD.

*Policy diffusion.* A handful of participants shared that the introduction of MAiD in Canada was influenced by the increased global implementation of assisted dying policies: “We were able to look to those other countries to see their experiences [with MAiD]. I think that was able to, in many ways, pave the path forward for MAiD to come here [to Canada] (P25).” Health leaders explained that policymakers in Canada gained valuable insights from examining international practices and available evidence regarding the impacts and ramifications of assisted dying. They considered the experience of several European countries and some American states to be an important influence in the process of MAID legalization and implementation in Canada.

*Healthcare system emphasis on a patient-centred care approach*. Participants described the introduction of MAiD as part of an increasing emphasis on a patient-centred care approach within healthcare services. Health leaders discussed a growing emphasis on patients’ “bodily autonomy” and “control” in their health decisions and care preferences. Participants explained that increasing patient empowerment, autonomy, and agency is aligned with providing MAiD through the healthcare system as an end-of-life option.

*Changes in societal and cultural values.* Many participants noted that the introduction of MAiD was aligned with a broader shift in societal and cultural values. They described social mobilization that led to legal changes with respect to same-sex marriage and abortion rights. Participants perceived public acceptance of other values to be related to the introduction of MAiD: “I feel like we got to a point in society where individual choice and human rights are really more acceptable (P15).”

### Health leaders’ attitudes towards the introduction of MAiD

Health leaders expressed wide-ranging attitudes towards the introduction of MAiD. Most participants were supportive of the introduction of MAiD in Canada. For example, they expressed: “I’m glad that MAiD has been legalized for people (P14)” and “I think C-14 was an incredible piece of legislation and ground-breaking (P9).” However, alongside their support, some participants also made clear that they disagreed with aspects of MAiD or proposed changes to the legislation. For example, some participants who were supportive of MAiD overall expressed concern that MAiD might be sought in some circumstances due to a lack of resources, or unavailability and inaccessibility of healthcare services.

Some participants expressed concern that MAiD may further perpetuate the vulnerabilities of marginalized populations: “I’m very respectful that [MAiD is] a legal right and that some people want this. I don’t question that, but I do see it being an easy-to-offer solution for people who don’t know what to do […] I worry that it’s become a tool that’s used more often just because of the lack of resources in our healthcare system (P6).” Other participants contended that there were sufficient safeguards and procedures in place to protect vulnerable populations.

Few participants expressed overall disagreement with the introduction of MAiD. However, some were uncomfortable with the idea of medical professionals playing a role in hastening death: “I do have questions about [MAiD] being seen as a medical procedure to end life because someone perceives that their life is not worth living anymore. Is that really the role of medicine? I’m not convinced of that (P37).” Likewise, a few participants held neutral attitudes and some shared similar sentiments as those who disagreed, but they did not definitively position themselves towards agreement or disagreement. Instead, they felt they needed to situate their attitudes on a case-by-case basis.

### Perceived factors that shaped health leaders’ attitudes towards the introduction of MAiD

Health leaders described four factors that influenced their attitudes toward the introduction of MAiD (Table 3).

*Personal illness experiences*. Health leaders described personal experiences that shaped their attitudes towards MAiD. For example, participants described how their own health circumstances and being confronted with mortality deepened their attitudes towards MAiD: “I actually went through treatment for breast cancer […] I would want it to be able to access it. So, when the legislation passed, there was a bit of personal relief to know that I had that option available to me personally (P24).” Other participants described their illness and end-of-life experiences with a family member, friend, or someone else within their social network.

*Professional experiences and identity.* Participants also described how their professional experiences informed their beliefs about MAiD. Some explained that their experiences led them to believe that aggressive treatments at the end of life may not be the optimal choice for all patients: “It was really my experiences as a nurse that put me on the path towards ethics […] Just let this person be, whatever time they have left, just let them spend it with the family [rather than them] spending with us poking and prodding […] when the outcome was still [that] these [patients] were not going to get better (P25).” Others described how their professional identity and beliefs about the purpose of medicine influenced their beliefs about MAiD: “I am a really big believer that physicians, ultimately what we do is we help people. […] We’re mostly givers and helpers, and so I actually see providing MAID as an extension of that work […] (P9).”

*Moral and religious beliefs.* Participants explained that their own moral and religious beliefs partially accounted for their attitudes towards the introduction of MAiD. Some discussed struggling to reconcile the differences between their personal values and the doctrine of their religion. Others described making a distinction between their religious beliefs and their professional responsibilities.

*Valence of patient autonomy and quality of life*. Health leaders described how their attitudes towards the introduction of MAiD were influenced by the value they placed on patient autonomy in decision-making and on respect for patients’ quality of life. They emphasized patients’ rights in making end-of-life care decisions and their support for patients dying with dignity. Participants also emphasized how important it was for patients to be fully engaged in their care and decision-making process, including decisions about their end-of-life care options: “We have the right to make decisions about everything in life except our death, you know? So why should we not be able to decide when and how we die? (P10).” Some participants further articulated their value for supporting patients’ quality of life and dignity in dying: “I think quality of life is […] more important than quantity of life (P14).”

## Discussion

This qualitative study sought to understand health leaders’ perceptions regarding the factors that shaped the introduction of MAiD in Canada, their attitudes towards the introduction of MAiD, and the factors they believed influenced their attitudes. Participants in this study were health leaders with different leadership roles (i.e., not exclusively healthcare providers) who held positions of influence in the healthcare system and were able to guide MAiD policy and practice. Understanding the perspectives of this group can provide insight into the complex dynamics and factors in Canada that led to the introduction of MAID and informed MAiD policy in Canada and can provide lessons for other countries considering introducing assisted dying. We discuss these findings and their implications for practice, policy research and development, as well as for medical ethics.

In this study, participants expressed wide-ranging and complex attitudes about the legalization of MAiD. While attitudes toward assisted dying are often presented in the public and professional discourse as binary, this study adds to the literature that highlights the important ‘muddy middle’ perspectives of health leaders [13–15]. A recent qualitative study conducted with healthcare providers in Spain identified four categories of attitudes towards MAiD: full support, conditioned support, conditioned rejection, and full rejection [24]. However, these ‘conditional’ categories do not fully encompass the complexity of the attitudes held by participants in our study. For example, some participants expressed overall agreement with the introduction of MAiD, while still raising concerns regarding the vulnerability of those seeking MAiD. Others indicated that their attitudes changed on a case-by-case basis. Some participants expressed discomfort with administering MAiD themselves but were in favour of ensuring appropriate access to MAiD based on human rights principles.

Developing policy and clinical practice guidelines for assisted dying has been at times divisive and challenging for policymakers [25]. The findings of our study suggest that some health leaders felt conflicted in that their ethical principles regarding the importance of patient autonomy seemed to be at cross-purposes with their views on the principle of beneficence in health care. Taking these and other principles into account is important, although it adds complexity to decisions at the level of clinical care, policy and legislation. Indeed, it is critical to consider how these ethical principles may be held simultaneously and appropriately tailored in their application, depending on the individual patient who is seeking MAID and their circumstances. As Konder and Christie [26] assert, there is potential for Canada to lead the way on protecting populations that may be vulnerable while still supporting autonomy and considering different sources of vulnerability.

This study’s participants described four broad influences on their attitude towards MAiD: personal illness experiences; professional experiences and identity; moral and religious beliefs; and valence of patient autonomy and quality of life. Similar to our findings, Jounou and colleagues [24] identified potential ethical issues that shape different positions of healthcare providers towards the practice of MAiD to include end-of-life care, religion, professional duty/deontology, and patient autonomy. Other research with healthcare providers has also highlighted the role of religion in shaping providers’ attitudes towards MAiD [27,28]. For example, Wong et al. [16] found that medical students who were willing to provide MAiD were less likely to practice a religion. However, Oliphant and Frolic [28] found in their study of health providers that religion was not always the defining determinant of providers’ involvement with MAiD, but participants for whom faith was important found a way to reconcile their participation in MAiD with their faith. Some participants in this study who described themselves as religious were nevertheless supportive of MAiD, which they credited to the value they placed on patient autonomy and quality of life. We found that professional and personal experiences of health leaders were consistently identified as factors shaping and reinforcing their attitudes toward MAiD, which is consistent with past Canadian studies with healthcare providers [27–29].

Overall, health leaders in this study described their attitudes toward MAiD as being influenced by personal motives or moral reasoning, rather than exclusively on established medical criteria or legal frameworks. This is not surprising, although it raises questions about the extent to which the personal values and moral beliefs of health leaders should be considered in the development of a policy. A similar tension between one’s professional responsibilities and personal values and beliefs may exist for clinical MAiD assessors/providers and other healthcare professionals who care for patients who receive MAID. Though completely impartial policymaking on this topic may not be possible, steps that can be taken to guard against the undue influence of personal beliefs and biases include the development of clear eligibility criteria and frameworks, guidelines for moral reasoning, an independent review process, and the incorporation of reflexive practices and transparency into policymaking.

Given the challenging, complex, and personal implications of this work, greater support to promote clarity and resilience of health leaders could be embedded into the system [30]. For example, the recently developed Canadian MAiD Curriculum for healthcare providers has a module on resilience and reflection [31, 32]. However, there are health leaders involved in MAID who work in sectors beyond healthcare, such as in government or academia. It may be helpful to expand the availability of supports to target these other health leaders, as well as to adapt informational, psychosocial or other supports to their differing roles and needs. Such support may be directed to health leaders individually, or as part of their profession or institution. These efforts should also be focused on addressing challenges associated with institutional objections to MAiD as this remains a persisting ethical concern of care recipients and healthcare providers [33].

A novel aspect of this study was examining health leaders’ perceptions about what led to the introduction of MAiD in Canada in 2016. Health leaders described six factors that they believed played a role in the introduction of MAiD: public advocacy and influence; judicial system and notable MAiD legal cases; political ideology and landscape; policy diffusion; healthcare system emphasis on a patient-centred care approach; and changes in societal and cultural values. Consistent with past studies, Buchbinder and Cain [34] also asserted that social movements and advocacy have shaped debates about the endoflife, and other research has acknowledged that legal cases, such as *Carter*, were critical in the emergence of MAiD in Canada [35]. There have been ongoing discussions regarding whether it is preferable for the courts or parliament to be responsible for legalizing MAiD [36–38]. The role of the public in shaping the momentum of policy is also noted by Preidel and Knill [39]. When conducting public deliberations related to MAiD it is important for policymakers to engage diverse members of the public and to recognize that the purpose of such discussions is not necessarily to reach a consensus, but to allow for sharing and clarifying perspectives of the public including those that are in conflict with each other [40].

The identification of these factors that led to the introduction of MAiD could support policy development in other jurisdictions contemplating introducing assisted dying. In Canada, these factors could guide continued policy development including changes in MAiD eligibility related to mental illness, mature minors, and advance requests [1–3]. Increasing public advocacy on assisted dying at times of political change and emphasizing MAiD as part of patient-centered care may also be influential in assisted dying policy development. In addition, it would be beneficial for health leaders to be better integrated into the comprehensive development, integration, and implementation of assisted dying [41]. Future research should examine what led (or did not lead) to the implementation of assisted dying in different jurisdictions using complementary methodologies and approaches [42]. Notably, it would be valuable to survey health leaders to understand the representativeness and importance of the perspectives and attitudes identified in this study.

### Strengths and limitations

This study’s strengths included the large number of interviews conducted with health leaders across Canada. Qualitative interviews of this kind can elicit a more nuanced response than survey responses and uncovered the wide-ranging and complex attitudes of health leaders concerning the introduction of MAiD. Another important strength of the study was a diverse research team with a range of academic disciplines, career stages, and personal backgrounds.

This study had several limitations. It was conducted with health leaders in Canada and therefore its generalizability to the development of assisted dying policies in other countries should be interpreted cautiously. In addition, data for this study were collected from 2021 to 2022, and health leaders’ attitudes could have changed and evolved over time. Our definition of health leaders was broad and included health leaders who may have had different impacts on the policy and practice of MAiD. The study sample size was limited and since many of the health leaders held multiple roles, we could not examine differences in health leaders’ perspectives and attitudes based on their role. It would be helpful in future research to identify and compare attitudes and opinions of health leaders with different leadership roles (e.g., academia, health professional, government, etc.). Finally, initial code development and analysis was conducted mainly by one reviewer (AY) though a larger team met weekly to discuss code development, review data, and provide input on thematic selection in order to diminish potential bias.

## Conclusion

This qualitative study explored health leaders’ attitudes towards MAiD and their perspectives on what shaped the introduction of MAiD in Canada. This study contributes to the growing literature on health leaders’ wide-ranging attitudes toward MAiD. Although their attitudes toward assisted dying are often presented as binary (either in favour or against), this study highlights the multifaceted perspectives of health leaders. It also identified the convergence of multiple factors that health leaders believed contributed to the legalization of MAiD in Canada. These findings can inform policy development in Canada where changes to MAiD eligibility are ongoing, and in other jurisdictions considering the legalization of assisted dying.

**Table 2:** Perceived factors leading to the introduction of MAiD

Theme name	Description	Example quotes
**Public advocacy and influence**	Participants observed how advocacy efforts by groups of individuals and organizations shaped public opinion and generated public support, which influenced the decision-making processes toward MAiD.	“[There have] been some lobby groups […] that ha[ve] become quite credible and has supported write-in campaigns, lobbying of MPs, and that probably would’ve supported the process [towards MAiD] (P38).” “There was a fair amount of advocacy from certain individuals and from certain organizations and a little bit from the public as well, and some public service people, like government, to consider the changes in the law so that people would have time to accept death or hastened death (P35).”
**Judicial system and notable MAiD legal cases**	Participants highlighted how the judicial system played a critical role in interpreting and applying laws, including important court opinions/cases that have influenced the formation of MAiD.	“I don’t think there was [a] strong [MAiD] movement here in Canada, but because there were individuals like Carter who took this case all the way to the Supreme Court […]. We’ve done it backwards compared to other countries, our government had to respond to those court decisions rather than developing a movement over time to kind of get the nuances […] It was individual cases like Rodriguez or Carter that started the process (P3).” “I guess that’s what Supreme Courts do though and it probably was because the people bringing this case [of MAiD] had become frustrated through the political change. The political change was going to go too slowly for the patients, the people, who wanted to have the hastened end-of-life and the avenue available to do them when your political change is too slow to go to the Court. So, it makes sense that they went to the Court (P32).”
**Political ideology and landscape**	Participants referred to the shift in ideologies and values within the Canadian political system around the time of MAiD introduction.	“I feel like [the Liberal Party of Canada] need[ed] to make sure that they’re providing what people want, and so they were able to make [MAiD] happen. I think we only got [MAiD] because the Liberals got in, we wouldn’t have had it if the Conservatives stayed in (P36).” “We’re a liberal society. It’s just the U.S. has a very conservative mindset in the judiciary [related to MAiD], we have a more liberal mindset (P22).”
**Policy diffusion**	Participants noted how the increasing development and implementation of assisted dying policies in countries outside of Canada shaped its introduction in Canada.	“We were able to look to those other countries to see their experiences [with MAiD]. I think that was able to, in many ways, pave the path forward for MAiD to come here [to Canada] (P25).” “We also had new evidence, empirical evidence […] [and] then from Oregon and Washington state, the Netherlands, [and the] Benelux countries that demonstrated that there was no slippery slope. They didn’t have that at the time of Rodriguez (P17).” “Internationally speaking, of course, by the time [Canada] got to our decision [in 2016], there had been several jurisdictions around the world that had already gone ahead with the legalization or decriminalization or some aspect of permitting [MAiD]. So, I feel like there was enough of an understanding of what’s happened in those jurisdictions for the Canadian courts to really get what the implications were of legalizing MAID here and what the potential trajectories could look like (P28).”
**Healthcare system emphasis on a patient-centred care approach**	Participants described the system-level transformation of the Canadian healthcare system on placing patients at the centre of the care decision-making processes, supporting greater patient autonomy and choice and ensuring their voices are heard and their choices are respected.	“…I think the same individuals who’ve been pushing for things like MAiD are the same ones who push for things like abortion access and ensuring that rights are protected, and various different areas related to human rights in general or human rights specifically related to healthcare, like bodily autonomy (P21).” “It’s not surprising to me that patients want to take back some of that control around their end-of-life experience. I mean, of course, patients are informed and have their choice around whether they’re doing treatment and what treatment and where they want to be, but it is still very much dictated by our healthcare system as to what options people have and how it’s presented (P27).”
**Changes in societal and cultural values**	Participants observed how societal changes towards sharing common values and acceptance of human rights within and outside of healthcare, shaped the movement towards MAiD.	“I feel like we got to a point in society where individual choice and human rights are really more acceptable. As a society, we’ve moved past things like no gay marriage and no abortion. And like all these other things that, I think 40 years ago, people felt really differently about, but societal norms have changed (P15).” “I think a general movement towards understanding that people’s own choices are their own choices. We seem to have moved through legalizing abortion or at least decriminalizing abortion and legalizing same-sex marriage and same-sex adoption and MAiD has come after all of that. Why that particular order? I don’t know, but I think it’s just a move towards realizing that people’s personal decisions are theirs and that there shouldn’t be any barriers to that (P24).”

**Table 3:** Perceived factors that shaped health leaders’ attitudes towards the introduction of MAiD

Theme name	Description	Example quotes
**Personal illness experiences**	Participants described personal experiences outside of their professional work, with family, friends, and neighbours related to the end of life. These experiences led them to further reflect on the value of MAiD.	“I actually went through treatment for breast cancer […] and at the time I remember thinking I wish MAiD was available because if this got worse, I would want it to be able to access it. So, when the legislation passed, there was a bit of personal relief to know that I had that option available to me personally (P24).” “I had one grandparent who died suddenly in her sleep, so there was no goodbye. There was no chance to have any sort of ending with loved ones […] I think about how with MAiD as an option, people would have that opportunity. Now here we are a few years in and seeing just how amazing those final moments and those goodbyes happen (P21).” “I had a neighbour who had brain cancer and died […]. He was a proponent of MAiD, [but] it was before MAiD [was available]. I remember his wife talking about how, like, if [MAiD] had been an option in his last weeks of life, he would have taken it because it would have allowed him to die with greater dignity. […] As soon as I heard his story, I was like, okay, I think this is an important component of care that should be offered to patients (P11).”
**Professional experiences and identity**	Participants emphasized how paid work or career experiences with end-of-life care and MAiD services shaped their attitudes towards MAiD.	“It was really my experiences as a nurse that put me on the path towards ethics […] [and] Just let this person be, whatever time they have left, just let them spend it with the family [rather than them] spending with us poking and prodding, and doing CPR, and hooking them up to all sorts of machines when the outcome was still [that] these [patients] were not going to get better (P25).” “I am a really big believer that physicians, ultimately what we do is we help people. That’s what physicians do. Once in a while, we diagnose, [and] a few times we even cure them. But most of the time all we really do is help people. We comfort people. We hold their hand when we can’t help people the best we can. We’re mostly givers and helpers, and so I actually see providing MAID as an extension of that work […]. It perfectly fits in with my background in […] medicine (P9).”
**Moral and religious beliefs**	Participants reflected on their own personal and moral beliefs toward MAiD within their religious contexts. This included a struggle between or reconciliation of their personal and religious beliefs, or rejection of their religious beliefs.	“As a Catholic, my religion would be against it. I also realize that professionally and I think morally I have to recognize the suffering of others and that others have different perspectives [and] different approaches to it. And that it is not my place to impose my personal views or those of anything I represent from a personal perspective on other people especially not in my roles on the palliative care unit […or] in creating federal policy (P31).” “I think within the religion that’s where the equation happens, [whereby] if you are deciding to go through with MAiD, I think a lot of people will look at that as you are committing suicide. I think that’s why I have been able to think about it a little bit differently, where I think that they’re two completely separate things, where even the support services and the resources out there for those who have suicidal thoughts are very different than the people who are interested to pursue MAiD (P14).”
**Valence of patient autonomy and quality of life**	Participants valued engaging the patients in healthcare decision-making, related to their rights to end-of-life care, dying with dignity, and having MAiD as a care option. This includes the importance of respecting patients’ desires to have a better quality of life.	“We have the right to make decisions about everything in life except our death, you know? So why should we not be able to decide when and how we die? You can decide everything else […] you can refuse chemo and you can decide not to go to ICU, and you can decide not to have CPR, but you have to have this prolonged lingering death (P10).” “I think quality of life is […] more important than quantity of life. I think if we can assess the mental capabilities of a person to make sure that they [have] the ability to make a decision, then I think that that should be respected. And that [the patients] are able to then choose the end in like a manner that’s dignified for them (P14).” “As a social worker, there are some ethically really challenging situations that we face when we’re working with patients who ask for MAiD, but at the end of it, it is their choice, and it is their decision. I think if we can provide the best care to them throughout their whole illness trajectory, and if this is how they want to die then we need to support them to do that (P15).”

## Electronic supplementary material

Below is the link to the electronic supplementary material.

Supplementary Material 1

## Data Availability

The qualitative data collected and analysed in this study are available from the corresponding author on reasonable request and if the request meets research ethics and governance criteria.
