# Supplementary material for: Health leaders’ perspectives and attitudes on medical assistance in dying and its legalization: a qualitative study
Source: BMC Med Ethics. 2025 May 6;26:57. doi: 10.1186/s12910-025-01208-2 (PMC12053847; doi:10.1186/s12910-025-01208-2)
Supplement: Supplementary file 1 — Supplementary Material 1 [file 12910_2025_1208_MOESM1_ESM.docx]

**Supplementary Material**

**Consolidated Criteria for Reporting Qualitative Studies (COREQ) Checklist**

| **Item** | **Topic** | **Guide Questions/Description** | **Reported on page no.** |
| --- | --- | --- | --- |
| **Domain 1: Research team and reflexivity** | | | |
| *Personal characteristics* | | | |
| 1 | Interviewer/facilitator | Which author/s conducted the interview or focus group? | Data Collection, p.8 |
| 2 | Credentials | What were the researcher’s credentials? E.g. PhD, MD | Author Information, p.1 |
| 3 | Occupation | What was their occupation at the time of the study? | Study Design, p.7 |
| 4 | Gender | Was the researcher male or female? | Study Design, p.7 |
| 5 | Experience and training | What experience or training did the researcher have? | Study Design, p.7 |
| *Relationship with participants* | | | |
| 6 | Relationship established | Was a relationship established prior to study commencement? | Data Collection, p.8 |
| 7 | Participant knowledge of the interviewer | What did the participants know about the researcher? e.g. personal goals, reasons for doing the research | Data Collection, p.8 |
| 8 | Interviewer characteristics | What characteristics were reported about the interviewer/facilitator? e.g. Bias, assumptions, reasons and interests in the research topic | Data Collection, p.8 |
| **Domain 2: Study design** | | | |
| *Theoretical framework* | | | |
| 9 | Methodological orientation and theory | What methodological orientation was stated to underpin the study? e.g. grounded theory, discourse analysis, ethnography, phenomenology, content analysis | Data Analysis, p.9 |
| *Participant selection* | | | |
| 10 | Sampling | How were participants selected? e.g. purposive, convenience, consecutive, snowball | Study Participants, p.7-8 |
| 11 | Method of approach | How were participants approached? e.g. face-to-face, telephone, mail, email | Data Collection, p.8 |
| 12 | Sample size | How many participants were in the study? | Data Collection, p.8-9 |
| 13 | Non-participation | How many people refused to participate or dropped out? Reasons? | Not reported due to word limit. Please see Shapiro et al (14) Appendix 1, Section 2 |
| *Setting* | | | |
| 14 | Setting of data collection | Where was the data collected? e.g. home, clinic, workplace | Data Collection, p.8 |
| 15 | Presence of non- participants | Was anyone else present besides the participants and researchers? | Data Collection, p.8 |
| 16 | Description of sample | What are the important characteristics of the sample? e.g. demographic data, date | Participants Characteristics, p. 10; Table 1 |
| *Data collection* | | | |
| 17 | Interview guide | Were questions, prompts, guides provided by the authors? Was it pilot tested? | Data Collection, p.8 |
| 18 | Repeat interviews | Were repeat interviews carried out? If yes, how many? | Not reported due to word limit. Repeat interviews were not conducted. |
| 19 | Audio/visual recording | Did the research use audio or visual recording to collect the data? | Data Collection, p.8 |
| 20 | Field notes | Were field notes made during and/or after the interview or focus group? | Not reported due to word limit. The interviewer made field notes after the interviews. |
| 21 | Duration | What was the duration of the interviews or focus group? | Data Collection, p.9 |
| 22 | Data saturation | Was data saturation discussed? | Data Collection, p.9 |
| 23 | Transcripts returned | Were transcripts returned to participants for comment and/or correction? | Not reported due to word limit. Transcripts were not returned to participants for comment and/or correction. |
| **Domain 3: Analysis and findings** | | | |
| *Data analysis* | | | |
| 24 | Number of data coders | How many data coders coded the data? | Data Analysis, p.9 |
| 25 | Description of the coding tree | Did authors provide a description of the coding tree? | Tables 2 and 3 |
| 26 | Derivation of themes | Were themes ident  ified in advance or derived from the data? | Data Analysis, p.9 |
| 27 | Software | What software, if applicable, was used to manage the data? | Data Analysis, p.9 |
| 28 | Participant checking | Did participants provide feedback on the findings? | Data Collection, p.9 |
| *Reporting* | | | |
| 29 | Quotations presented | Were participant quotations presented to illustrate the themes / findings? Was each quotation identified? e.g. participant number | Results, p.10-17 |
| 30 | Data and findings consistent | Was there consistency between the data presented and the findings? | Results, p.10-17 |
| 31 | Clarity of major themes | Were major themes clearly presented in the findings? | Results, p.10-17 |
| 32 | Clarity of minor themes | Is there a description of diverse cases or discussion of minor themes? | Results, p.10-17 |
